# Implications and issues related to familial pancreatic cancer: a cohort study of hospitalized patients

**DOI:** 10.1186/s12876-016-0421-8

**Published:** 2016-01-15

**Authors:** Martina Mughetti, Lucia Calculli, Anna Maria Chiesa, Federica Ciccarese, Odeta Rrusho, Raffaele Pezzilli

**Affiliations:** Department of Diagnostic and Preventive Medicine, Sant’Orsola-Malpighi Hospital, Via Massarenti 9, 40138 Bologna, Italy; Pancreas Unit, Department of Digestive System, Sant’Orsola-Malpighi Hospital, Via Massarenti 9, 40138 Bologna, Italy

**Keywords:** Clinical assessment, Familiality, Pancreatic cancer, Pancreatic mucinous neoplasms

## Abstract

**Background:**

The surveillance of subjects at risk of pancreatic cancer is restricted to clinical research; the incidence of familial pancreatic cancer needs to be better established. Thus, we aimed to evaluate the frequency of familial pancreatic cancer in a population of hospitalized patients with pancreatic cancer.

**Methods:**

A retrospective study based on the hospital charts of patients discharged with a diagnosis of pancreatic cancer. One hundred and eighty-seven patients or their relatives were called for a phone interview.

**Results:**

There were 97 males (51.9 %) and 90 (48.1 %) females. The overall mean ± SD age was 67.3 ± 11.8 years; the age of males was similar to that of females (*P* = 0140). The mean size of the tumors found was 36.3 ± 17.4 mm (range of 5–110 mm); it was related to gender but was not related to the site of the tumor or the age of the patient. Regarding genetic diseases, three females (1.6 %) had familial adenomatous polyposis; three patients (1 male and two females) (1.6 %) had at least one relative with pancreatic cancer whereas only one 80-year old male patient (0.5 %) had two relatives affected by pancreatic cancer (the mother had died at the 65 years of age and the brother had died at 75 years of age).

**Conclusions:**

The frequency of familial pancreatic ductal adenocarcinoma is small, but its importance, from the point of view of early diagnosis, is not negligible and patients with a risk of familial cancer merit an appropriate clinical follow-up.

## Background

Pancreatic cancer is the fourth leading cause of cancer death in Western countries [1], and its incidence and mortality is increasing [2]. Its incidence varies widely in different populations, suggesting the important role of environmental risk factors, lifestyle and genetic factors [3]. Given the low incidence of pancreatic cancer, screening in the general population is still under debate due to the limited number of cases [4]. However, the presence of a group of individuals having an increased risk of developing pancreatic cancer has been defined; people who have a genetic or somatic condition associated with a 5–10 fold increased relative risk should be involved in a surveillance program for prevention or early detection, with the aim of reducing mortality by means of a precocious surgical resection [5]. Surveillance programs for people at risk have so far been generally limited to clinical trials, but acquiring a deeper understanding of this complex field and the growing need for treatment of patients with precancerous lesions using a minimally invasive approach, becomes increasingly necessary [6]. Pancreatic preventive surgery should be performed in centers with a high level of expertise in order to reduce the complications related to this type of intervention [7]. Data regarding the incidence of familial cancer in the population are not well established. The incidence in Northern Italy has recently been estimated at 0.6 % of pancreatic adenocarcinomas [8].

The purpose of this study was, therefore, to assess the frequency of familial pancreatic cancer in a population treated in our Hospital. The ancillary endpoints were to assess other risk factors depending on the lifestyle of the population, such as smoking, alcohol, coffee, overweight/obesity, diabetes and also other types of cancer, both synchronous and metachronous, associated with pancreatic cancer.

## Methods

This was a retrospective study based on hospital discharge records regarding codes 157.0 and 157.1 of the Italian Classification ICD-9-CM [9] from May 2011 to December 2013. All patients were selected from St. Orsola-Malpighi Hospital in Bologna at first presentation for diagnostic and therapeutic evaluation for pancreatic cancer.

**Fig. 1:**
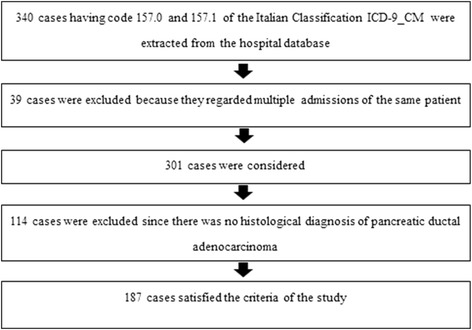
Flow chart reporting how the selection of patients enrolled in the study was made

As reported in Fig. 1, 340 cases were present in the hospital database of whom 187 patients were selected and constituted our study population; these patients or their relatives were contacted by telephone on March and April 2014 and 148 patients (79.1 %) were still alive and they were able to give information. The data requested by phone interview were those which were lacking in the hospital charts of patients, such as smoking, coffee and alcohol habits and clinical data (diabetes, presence of pain, etc.) and was carried out by an experienced physician (RP).

All radiologic studies were reviewed by a dedicated radiologist (LC). Regarding the imaging techniques computed tomography (CT) and magnetic resonance imaging (MRI) with or without magnetic resonance cholangiopancreatography (MRCP) according to the protocols in use in our hospital. Endoscopic ultrasound (EUS) was performed if clinically appropriate.

Since this was a retrospective study, the “*post hoc*” sample size was calculated. Based on an incidence of familial pancreatic cancer ranging from 0.4 to 1 % of all diagnosed pancreatic cancer (mean 0.6 %), taking into account an alpha error of 0.05 and a cumulative incidence of familial pancreatic cancer of 2.1 % in our population of 187 patients, the power of the dichotomous endpoint (familial pancreatic cancer vs. non familial pancreatic cancer) for one sample study was 64.6 %; thus, this latter figure means that the study was not underpowered [10].

### Ethics

The study was approved by the Senior Staff Committee of the Department of Radiology of the University of Bologna and was carried out in accordance with the Helsinki Declaration of the World Medical Association. All study participants gave informed consent.

### Statistics

The descriptive statistics applied were means, standard deviations (SDs) and ranges as well as absolute and relative frequencies. Non parametric statistics were applied: the Mann Whitney *U* test and the Pearson chi-squared test were applied where appropriate. All statistical evaluations were carried out by running SPSS version 13.0 for Windows. Two-tailed *P* values less than 0.05 were considered statistically significant.

## Results

Of the 187 patients, 97 were male (51.9 %) and 90 (48.1 %) were female. The average age of the entire study population was 67.3 ± 11.8 years (range 31-88 years). The average age of the males was 66.1 ± 11.4 and that of females was 68.5 ± 10.7 (*P* = 0140). The body mass index (BMI) in the entire population was 24.7 ± 4.1 kg/m2 (range 15.6 to 44.6 kg/m2). The BMI was similar (*P* = 0.659) in males (24.5 ± 3.5) and in females (25.0 ± 4.7). According to the World Health Organization (WHO) classification [11], 5.3 % were underweight, 50.0 % were normal weight, 38.2 % were overweight and 6.6 % were obese. There was no difference (*P* = 0.735) in the distribution of the BMI according to the various classes of BMI related to the sex of the patients. Regarding alcohol habit, 45 (24.1 %), of the 187 patients studied were alcohol users (28 males and 17 females) and the difference between drinkers, according to sex, was not statistically significant (*P* = 0.077). Ninety-three patients were smokers (49.70 %), and male smokers (61, 62.9 %) were statistically more numerous than female smokers (32, 35.6 %; *P* < 0.001). One hundred and two patients (54.5 %) were coffee drinkers, 54 males (52.9 %) and 48 females (47.1 %; *P* = 0.771). Regarding diabetes mellitus, one male patient (0.5 %) had diabetes mellitus type I, 61 (38 males and 23 females, 32.6 %) had diabetes mellitus type II and three (one male and two females, 1.6 %) had diabetes mellitus found at time of the diagnosis of pancreatic cancer. The frequency of males with diabetes was similar to that of females (*P* = 0.152). With regard to the localization of the pancreatic tumor, the tumor was localized in the head of the pancreas in 112 patients (59.9 %), in the body in 16 patients (8.6 %), in the tail in 21 patients (11.2 %), in the head-body in 17 patients (9.1 %) and in the body-tail in 21 (11.2 %); there were no differences regarding sex. Intraductal papillary mucinous neoplasms (IPMNs) were found to be associated with the pancreatic mass in 15 patients (8.0 %, 7 males and 8 females, *P* = 0.788) and it was of multifocal type in 3 patients (1.6 %). Eleven (5.9 %) of the 187 patients had a synchronous carcinoma and 24 (12.8 %), metachronous cancer with no difference between males and females (*P* = 0.540 and 0.283, respectively). Among the eleven patients having synchronous and the 24 having metachronous cancers, the primary cancer does not have a hint to a hereditary diagnosis.

The size of the mass were 36.3 ± 17.4 mm (range of 5–110 mm). The dimensions were related to sex (males: 39.4 ± 18.4, females 33.0 ± 15.6) (*P* = 0.039) but were not related to the location or age of the patients. Of the 187 patients, 130 (69.5 %) had a preoperative diagnosis, 91 (48.7 %) were diagnosed during surgery and 24 (12.8 %) were diagnosed both before and after surgery. Of the 115 patients undergoing surgery, 91 had resection surgery (79.1 %) and 24 had derivative surgery (20.9 %).

### Imaging techniques

One hundred and eighty-one of the 187 patients (96.8 %) underwent contrast-enhanced multidetector computed tomography, 6 (3.2 %), magnetic resonance imaging, 19 patients MRI and MRCP (10.2 %) and 60 (33.1 %) with EUS. Of the 6 patients who did not undergo the CT, three patients had undergone MRI, two patients MRI with MRCP and one patient EUS.

### Familiality

Regarding genetic diseases, three female patients (1.6 %) had familial adenomatous polyposis (FAP). In addition, three patients (1 male and 2 females) (1.6 %) had at least one relative with pancreatic cancer (two sisters, one daughter) while only one patient 80 years of age (0.5 %) had two family members (mother 65 years of age and brother 75 years of age) who had been diagnosed with pancreatic cancer. Thus, familial pancreatic cancer was found in 2.1 % of our population (3 subjects with FAP and one subject having with two relatives who died from pancreatic cancer).

## Discussion

This study states the reasonable hypothesis that a significant proportion of patients presenting with pancreatic cancer have a familial incidence such which this retrospective study identified.

It has been reported that in a study involving 570 families and including 9204 relatives whose probands were 3- to 5-fold more often heavy smokers than the general population, and 9.3 % of them reported a positive family history of pancreatic cancer; in addition, in first-degree relatives, only mortality from pancreatic cancer was significantly increased and the lifetime risk of dying from pancreatic cancer was 4.1 % for the relatives of all probands, and was 7.2 % for the relatives of probands who developed disease prior to 60 years of age [12]. These data suggest that genetic susceptibility to pancreatic cancer may be attributable to moderate- to low-penetrance genes. Several guidelines have also been developed to manage these high-risk individuals [13, 14] and registries have been established [15, 16] in order to better investigate this topic. However, there are no data regarding the incidence of familial pancreatic cancer in a real world of hospitalized patients. Thus, our study retrospectively evaluated all patients suffering from pancreatic cancer treated in Sant’Orsola-Malpighi Hospital in a period of three years. Regarding the primary endpoint of the study, only 1.6 % of patients had at least one relative with pancreatic adenocarcinoma while 0.5 %, had a family history of familial adenocarcinoma. Pancreatic cancer has a high mortality rate but its low incidence, together with the inability to arrive at a diagnosis using low cost imaging methods, does not seem to justify the execution of screening the general population, and it is therefore important to try to intervene first on the risk factors, such as smoking, coffee, alcohol and diet [17]. Based on the observations made in this study with respect to cases of familial pancreatic cancer, the introduction of screening at least 10 years before the age of the youngest family member affected would be recommended with the use of imaging methods, such as MRI and/or CT so that early intervention can be carried out with resection surgery. It should also be noted that our percentage of patients who should be screened is similar to that of another Italian study [8] and it would be appropriate, given the low number of patients to be followed, to abandon the current lack of interest regarding pancreatic cancer, at least in the familial form [18]. Only one of our patients was diagnosed at an advanced age and had never undergone a radiological investigation for screening as recommended by the Italian guidelines for familial cancer [13]. In addition, 1.6 % of patients had a tumor associated with FAP; this issue merits better investigation in clinical practice because screening for colorectal carcinoma may add useful information in selecting patients with FAP, and these patients should also be investigated and followed for the development of pancreatic cancer [19]. It is possibly that the future development of diagnosing the predisposition of familial pancreatic cancer is to evaluate genomic modifications; in fact, it has been recently reported that prevalence of mutations among familial pancreatic cancer probands was 1.2 % for BRCA1, 3.7 % for BRCA2, 0.6 % for PALB2, and 2.5 % for CDKN2A [20]. In addition, the same authors reported that familial pancreatic cancer probands carry mutations in the four genes with a significant more high frequency (8.0 %) than nonfamilial pancreatic cancer probands (3.5 %) [20]. On the other hand, other authors have been also reported that eleven pathogenic mutations have identified (3 in ATM, 1 in BRCA1, 2 in BRCA2, 1 in MLH1, 2 in MSH2, 1 in MSH6, and 1 in TP53) and that the prevalence of mutations was 3.8 %; the carrier status was associated significantly with breast cancer in the proband or first-degree relative, and with colorectal cancer in the proband or first-degree relative, but not family history of pancreatic cancer, age at diagnosis, or stage at diagnosis [21]. Thus, we need of further studies on this topic but genetic testing of multiple relevant genes in probands with a positive family history of cancer is reasonable.

Other data from our study confirmed what is already known, i.e. that approximately half of the study population smoked [20], about 50 % habitually consumed coffee [20] and less than a quarter of the patients (24.1 %) drank alcohol [22]. It has been suggested that obesity is a risk factor for pancreatic cancer [23], but our data seemed to confirm that, in the majority of cases (50.0 %), Italian patients were of normal weight, 38.2 % were overweight and only 6.6 % were obese. Probably, the diet of the Italian population is different from the American diet so the risk related to obesity does not appear to be evident [24]. Our data were in line with those of the literature regarding the location of pancreatic cancer; in fact, this tumor is most frequently localized in the head of the pancreas (60 %) [25], and the size of the mass, greater than 3 cm, means that the diagnosis is unfortunately still too late [26]. An interesting datum was that IPMNs were found to be associated with a pancreatic mass in 8 % of the patients and was multifocal in 3 patients. This indicated that pancreatic adenocarcinoma probably arises from the degeneration of an unrecognized mucinous tumor [27]; hence, the importance of a proper diagnosis and follow-up of mucinous cystic lesions [28]. Another interesting fact is that of the 187 patients studied, approximately 6.0 % had a synchronous carcinoma and 13 % metachronous cancer; these patients require a different clinical approach which has not yet been defined. Even if our study suffers from the limitations of retrospective studies, this is the first study, however, which evaluated the incidence of familial pancreatic cancer in an unselected hospitalized population; the post-hoc analysis revealed that the population study was not unpowered. In addition, it should be pointed out that only 62 % of the patients studied (187/301) had a correct Classification ICD-9-CM; much effort should be made to improve the correct application of Classification ICD-9-CM as also suggested by the Italian Association of Medical Oncologists [29]. One problem of studies regarding familial pancreatic cancer is the method used to assess the family history of these subjects. PancPro is a Bayesian modeling framework used to assess the pancreatic cancer risk of patients with a family history of pancreatic cancer [30]. However, in this study, information was collected using a questionnaire and no natural language processing system techniques. Using PancPro, it has been reported that the sensitivity of finding a subject with a family history of pancreatic cancer is 93 %, the positive predictive value is 97 % when family history is extracted, but this method considers only family histories [31] and does not extract specific family members or classify family members as primary, secondary and unknown relatives [32]. An algorithm has recently been proposed based on the Unstructured Information Management Architecture (UIMA) framework consisting of section segmentation, relation discovery and negation detection [32]. This system was evaluated using data from two institutions, and the results showed that rule-based natural language processing approaches for specific information extraction tasks are portable across institutions; however, customization of the algorithm regarding the new dataset improves its performance and this may increase the possibility of detecting the familial origin of pancreatic cancer.

## Conclusions

In conclusion, the frequency of familial pancreatic ductal adenocarcinoma in a non-selected series of patients, even if retrospective, is low, but its importance from the point of view of early diagnosis is not negligible and patients with familial cancer risk require adequate follow-up [17]. To really increase the possibility of finding subjects having familial pancreatic cancer, a new approach based on the Unstructured Information Management Architecture (UIMA) framework [32] should be tested in a large multicenter study. Finally, it has been demonstrated that screening for familial pancreatic cancer is cost-effective [33] and close cooperation between practitioners and referral pancreatological units may be of benefit both for the patient and for better economic resource allocation regarding health welfare.

## Abbreviations

ATM: ataxia telangiectasia mutated gene
BMI: body mass index
BRCA1: breast cancer type 1 susceptibility protein
BRCA2: breast cancer type 2 susceptibility protein
CDKN2A: cyclin-dependent kinase inhibitor 2A
CT: computed tomography
EUS: endoscopic ultrasound
FAP: familial adenomatous polyposis
IPMN: intraductal papillary mucinous neoplasm
MLH1: mutl homolog 1
MRCP: magnetic resonance cholangiopancreatography
MRI: magnetic resonance imaging
MSH2: MutS protein homolog 2
MSH6: MutS protein homolog 6
PALB2: partner and localizer of breast cancer 2
TP53: tumoral protein 53 gene
UIMA: unstructured information management architecture
